# Intelligent Intensive Care Unit: Current and Future Trends

**DOI:** 10.1007/s44231-023-00036-5

**Published:** 2023-05-16

**Authors:** Zhi Mao, Chao Liu, Qinglin Li, Yating Cui, Feihu Zhou

**Affiliations:** Department of Critical Care Medicine, The First Medical Centre, Chinese PLA General Hospital, Beijing, 100853 China

**Keywords:** Intelligent intensive care unit, Internet of things, Artificial intelligence, Robots, Big data

## Abstract

There is a growing demand for intensive care units, but there is a relative shortage of medical staff. Intensive care work is heavy and stressful. Optimizing the working conditions and processes of the intensive care unit is of great significance for improving the work efficiency and the level of diagnosis and treatment in the intensive care unit. The intelligent intensive care unit is a new ward management model gradually developed on the basis of modern science and technology such as communication technology, internet of things, artificial intelligence, robots, and big data. Under this model, the potential risks caused by human factors are greatly reduced, and the monitoring and treatment of patients has been significantly improved. This paper reviews the progress in related fields.

## Introduction

With the aging of the world and the improvement of medical conditions, more and more hospitals have set up comprehensive intensive care units, and some specialties have set up specialized intensive care units separately, to meet the treatment needs of more and more critically ill patients. A survey in the United States shows that there are more than 6300 ICUs in 3200 acute-care hospitals in the United States, with a total of 94,000 intensive care beds. However, critical care medicine places higher demands on the competence and experience levels of doctors and nurses. Patients in the intensive care unit are critically ill and unstable, often changing within a short period of time. Moreover, there is a large amount of equipment in the intensive care unit that is in operation for a long time. At the same time, the number of doctors and nurses in intensive care units has not kept pace with the number of patients, resulting in a shortage of manpower in many intensive care units [1]. Doctors and nurses have heavy workload, high occupational pressure, and are often in a state of exhaustion. It is easy to ignore the changes in the patient’s condition, and even lead to untimely treatment, which affects the work quality and treatment effect of the intensive care unit. In this case, on the one hand, the number of doctors and nurses should be increased, but at the same time, the labor cost will be significantly increased. On the other hand, the layout of intensive care units should be reasonably planned, and the work process should be optimized and standardized, so as to improve work efficiency and reduce the incidence of mistakes.

Smart ward is a new concept and design gradually formed in recent years. By adopting modern scientific and technological means, it can timely discover and solve problems in the management of inpatients, improve efficiency, reduce medical service errors caused by human factors, optimize the allocation of human resources, and optimize the use and monitoring of instruments and equipment. At present, the technologies integrated in smart wards mainly include: 5G communication technology, Internet, Internet of Things, artificial intelligence (AI), big data analysis, etc. As before, patients in the intensive care unit are in critical condition and have many instruments and equipment, which has more urgent and higher requirements for intelligence. In recent years, research on intelligent intensive care units has gradually increased. At present, the most research is in the setting of intensive care unit and intelligent monitoring and alarm, and there are also some researches in risk prediction. This study summarizes the related research on intelligent intensive care unit, in order to sort out the overview and progress of the current related research.

## Intelligent Intensive Care Unit

### The Definition and Composition, Function, Characteristics and Value of Smart Ward

At present, smart ward is still a relatively new name, and there is no clear, unambiguous definition. It is generally believed that smart ward is an important part of smart medical care. Smart medical care refers to the application of the most advanced Internet of Things technologies, such as the Internet, cloud computing, AI intelligence, big data and wireless sensors, etc., through the establishment of a medical information platform in the health record area, to realize the connection between patients and medical personnel, medical institutions, and medical equipment. The interaction between them gradually realizes informatization [2]. Smart hospitals are the main body of intelligent medical care, which can integrate various software and hardware to improve the efficiency of hospital services and management [3]. On this basis, the smart ward integrates the above technologies in the ward to achieve the purpose of intelligent management of patients, equipment, and ward software and hardware [4]. Smart ward is the core and focus of smart healthcare. In the smart ward, various technologies can monitor the vital signs and physiological parameters of patients in real time, analyze the data, and choose whether to trigger an alarm to send a reminder to doctors or nurses [5]. In the smart ward, appropriate technology can also carry out treatment or adjuvant treatment [6].

### The Definition and Composition Function, Characteristics and Value of Intelligent Intensive Care Unit

As the name suggests, the intelligent intensive care unit is based on the traditional intensive care unit, adding related technologies and facilities, so that the ward as a whole reaches a certain level of intelligence. Regionally, the intelligent intensive care unit includes ICU work area, functional support area and living area [7]. In these areas, through integration, the ICU can provide intelligent management of space and environment, intelligent management of instruments and equipment, intelligent service and management of patients, and services for patients include diagnosis, treatment, monitoring, remote consultation, and visits [7].

**Fig. 1:**
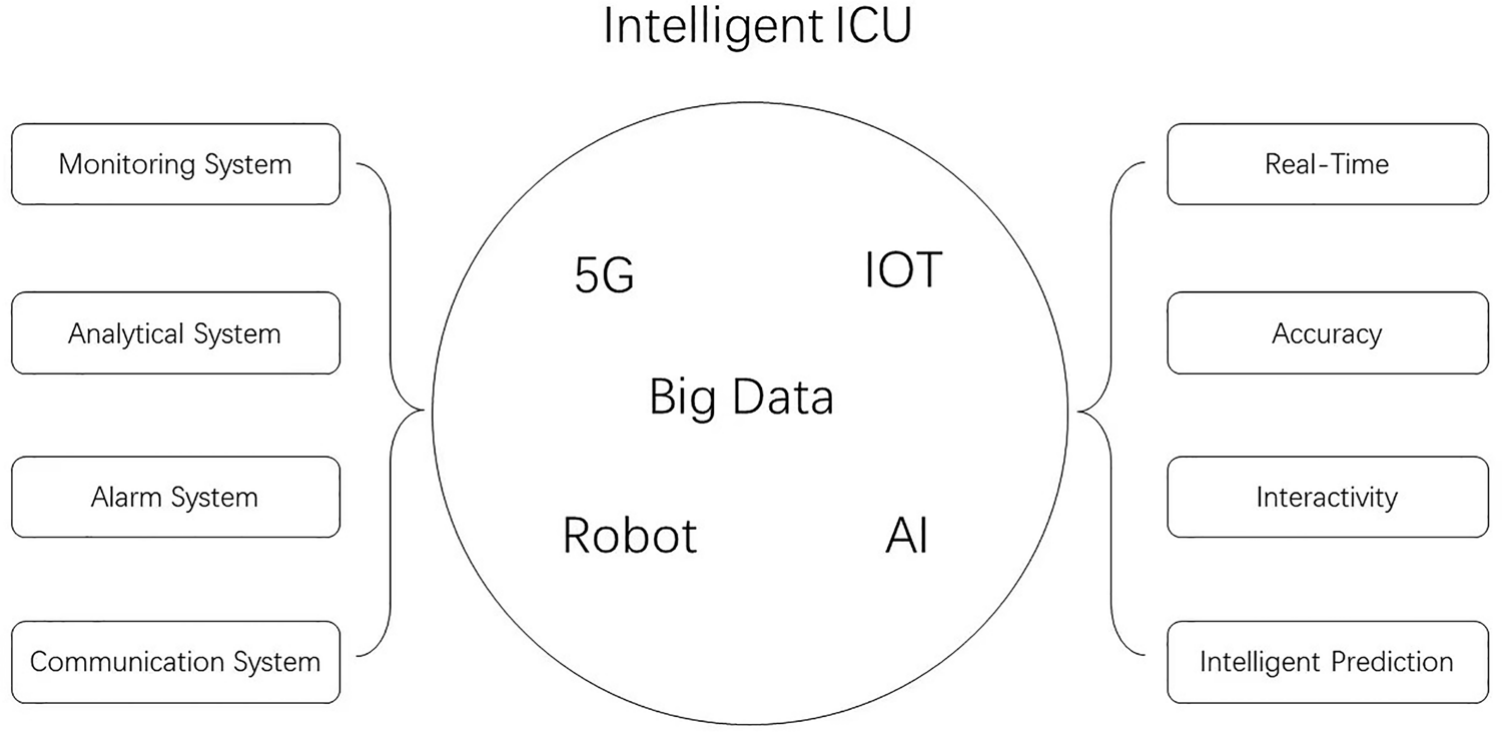
Composition function of intelligent ICU

In terms of systems, an ideal intelligent intensive care unit consists of the following parts: a monitoring system, which consists of a detection terminal and an analysis system. The detection terminal can detect the vital signs of patients and various important indicators, the operating status of instruments and equipment, indoor environmental parameters, etc.; the analysis system, which can analyze the data collected by the detection system, and combine the big data and the built-in parameter range to analyze the indicators; alarm system, the system will alarm and prompt for abnormal indicators, and can send an alarm to the medical staff or the central control room through different sounds, different warning light colors or alarm codes; communication system, this system is wired/wireles, complete the information transmission. Within and between these systems, technologies such as the Internet of Things, 5G, and AI are integrated. Through intelligent upgrading, several goals can be achieved for intensive care medical services: first, real-time detection of changes in the patient’s condition at the first time; second, accuracy, based on big data and AI, the changes in indicators and the abnormal value is judged, and different levels of alarms are given; third, the interactivity, medical staff or other staff can remotely adjust the parameters of the instrument and equipment, adjust the drug delivery speed, and modify the monitoring and analysis results, etc.; fourth, intelligent prediction, based on a large amount of real-time data, deep learning and AI, the system can make real-time judgments on the severity and prognosis of the disease. Through these effects, the efficiency and effectiveness of intensive care work can be significantly improved. (Fig. 1).

## Related Technologies Used in Intelligent Intensive Care Units

### 5G Communication Technology

Communication technology is the basis for realizing a certain area of intelligence, and 5G technology is the basis for real-time communication. 2019 is the first year of commercial use of 5G technology. Compared with 4G technology, the characteristics of high speed, low latency and large capacity of 5G technology can better ensure the effective application of people-to-people, people-to-thing, and thing-to-thing connections, and become the important technical support for further development of smart wards [8, 9]. For example, in recent years, studies have shown that remote robotic surgery can be carried out through 5G technology, so that patients at a distance can enjoy the medical services of top experts [10–12]. In the intensive care unit, repeated inspections and judgments are often required. For patients with complex conditions, it is often difficult for on-site doctors to immediately identify them. 5G technology can enable experts to remotely guide the inspection [12]. At the same time, the amount of data for inspection and related index parameters is large. To achieve real-time transmission and interaction of large amounts of data, fast signal transmission must be supported. However, the benefit of 5G technology in ICU has not been fully elucidated. Further investigation is warranted to observe the application and strength of 5G in intensive care medicine.

### Internet of Things (IoT)

IoT technology is widely used in intensive care units. Some studies have designed a hospital ICU emergency equipment alarm management system based on the Internet of Things technology, which integrates the functions of patient vital signs status display, real-time display of equipment operating status, abnormal alarm, video communication, and mobile APP. While providing the required patient data to guide medical decision-making, it implements hierarchical processing for instrument failure alarms and abnormal patient monitoring alarms, supports personalized alarm settings for patient monitoring, avoids unnecessary clinical interference, improves monitoring work efficiency, and ensures nursing care safety [13]. There are also studies that propose a management plan for medical equipment in the intensive care unit based on the Internet of Things, including three equipment interconnection methods based on private protocols, OpenICE standards, and Benelink modules, as well as a data platform construction plan. The author believes that the device interconnection solution based on the OpenICE standard provides a set of system solutions for device interconnection and interoperability, including the underlying device communication protocol, network architecture and top-level applications, and its integrated clinical application environment also considers medical device data and data. The matching of patient electronic medical record information represents the development direction of the Internet of Things construction of medical equipment in the future [14]. Liang Juan et al. proposed a remote monitoring and visiting system based on radio frequency identification (RFID) technology to provide patients with a safe, high-quality and recovery-friendly medical environment for visiting needs of the patient's family [15]. Zhu and others analyzed the characteristics of IoT terminal nodes based on ICU equipment, and established a data interaction management platform based on alliance chain security authentication and access control. Through this platform, data can be displayed and shared by third-party systems. Blockchain storage is used in ICU monitoring management to monitor patients' vital signs in an all-round way [16]. In a word, based on the Internet of Things technology, especially in the context of this new crown epidemic, it can effectively improve the efficiency of the management of patients and equipment in the intensive care unit, and facilitate multi-party interactive communication [17–19].

### AI

The research of AI in the medical field is very extensive, but the main application areas are in diagnosis, including imaging diagnosis, pathological diagnosis, laboratory diagnosis, etc. The application in the intensive care unit is also gradually emerging. Compared with diseases such as hypertension and diabetes, many interventions in the ICU lack evidence-based medical evidence, or the level of clinical evidence is not high. AI can analyze big data through algorithms, learn data laws, and make decisions and predictions on real events. In particular, the establishment and use of the medical information mart for intensive care (MIMIC) database in recent years has provided the basis for big data [20]. Based on this data, using AI methods, a large numbers of research results have been obtained. For example, some researchers have analyzed data and found that transthoracic echocardiography can reduce the mortality rate during ICU hospitalization in patients with sepsis [21]. Johnson and Krajnak et al. respectively established models with the goal of predicting ICU mortality, which can detect potential risks earlier and more accurately [22]. The method of Kho et al. can identify high-risk patients who may experience changes in their condition from electronic medical records [23]. Churpek et al. established an accurate prediction model of the risk of sudden death in patients during ICU transfer using logistic regression analysis based on hospitalization data of 250,000 patients. Therefore, using AI, based on the existing big data and research results, the specific patients in the ICU can be judged the risk of prognosis. A recent study showed beneficial effect of using the automated alerting system in the management of sepsis. Zhang et al. in their study found a beneficial effect of the alerting system in reducing mortality in septic patients. However, they also found that the automated alerting system shows less beneficial effects in the intensive care unit (RR 0.90; 95% CI 0.73–1.11) than that in the emergency department (RR 0.68; 95% CI 0.51–0.90) and ward (RR 0.71; 95% CI 0.61–0.82). One possible reason is the significant heterogeneity in enrolled studies concerning the study setting, design, and alerting methods in this study (24 Zhang Z, Chen L, Xu P, Wang Q, Zhang J, Chen K, Clements CM, Celi LA, Herasevich V, Hong Y. Effectiveness of automated alerting system compared to usual care for the management of sepsis. NPJ Digit Med. 2022 Jul 19;5(1):101. https://doi.org/10.1038/s41746-022-00650-5. PMID: 35854120; PMCID: PMC9296632.)

### Robots

In the ICU, robots can perform a variety of functions. Some studies have shown that doctors can conduct ward rounds remotely through on-site robots, especially in relatively remote small medical institutions, and can use robots to connect with remote experienced experts to diagnose and treat critically ill patients [1, 24]. In studies such as Murray, experts can control a remote robot through a laptop and a handle, and can issue instructions to move the robot around the ward, observe various parameters of monitors and ventilators, auscultate the heart and lungs, examine pupils, and communicate with patients, families, and medical staff [1]. The researchers believe that in this way, patients and medical staff can communicate with experts more quickly, balance medical resources between regions, and improve the quality of patient care [1, 24, 25], reduce ICU costs and ICU stay [24], and also improves patient safety [26], especially during the COVID-19 outbreak, the risk of exposure of healthcare workers can be greatly reduced through robotics or similar remote-control systems [27, 28].

### Big Data

Big data is the foundation of AI [29, 30], and is also the foundation of critical care medicine research [21, 31–33]. The establishment of a big data system in an intensive care unit often requires four main components: the connection between instruments and equipment, the internal information exchange and transmission system, the control center, and the server database, of which the connection with the monitoring equipment is the foundation [34]. Frontline clinicians should be at the heart of this dynamic learning system, fully supported by engineers, to collaboratively translate everyday problems into strategies for database querying, modeling, and analysis [35]. Through the analysis based on big data, the prognosis judgment, risk prediction, classification and grading of patients can be realized, to ultimately improve the outcome of patients [36–38].

The characteristics and applications of the above five technologies are summarized in Table 1.

**Table 1:** Modern technologies applied to intelligent intensive care units

Technologies	Function	Location	Clinical significance
5G	High speed, low latency, large capacity	The whole process, each system	Other technical role basis
IoT	Instruments and equipment are fully networked	Mainly in the ward, and related departments and departments	Improve patient management efficiency and hardware facility management efficiency
AI	Continuous and efficient learning	Mainly in the ward, monitor, diagnose and predict patients, monitor and alarm equipment	Improve the effect of diagnosis and treatment, optimize the performance and management of instruments and equipment
Robot	Stable, anti-risk, high repeatability	Mainly in wards and related functional areas	Save manpower, reduce the risk of exposure of medical staff, and improve service stability
Big Data	Evidence is reliable and representative	diagnosis and treatment of disease	Facilitate clinical research, assist diagnosis and treatment

*AI* artificial intelligence, *IoT* internet of things

## The Future Trends of Intelligent Intensive Care Unit

### Accurate Monitoring of Patient Condition and Judgment Alarm

The central processing system of the intelligent intensive care unit collects various important physiological data detected from the terminal. According to the normal value range in the memory and the built-in alarm threshold, artificial intelligence analyzes and determines whether it is abnormal, dangerous level, and triggers different alarm signals to achieve accurate and graded alarm.

### AI and Robot-Assisted Therapy and Nursing

In the intelligent intensive care unit, AI analyzes various data of patients, assists medical staff in diagnosing and judging the condition, conducts deep learning based on big data, and proposes inspection and treatment suggestions based on specific cases. Medical staff can refer to AI. It is recommended to adjust and optimize the treatment of patients in a timely manner. At the same time, robotic systems, especially those integrated with AI, can assist doctors and nurses to perform treatment operations on patients, such as assisting in real-time various punctures, drug distribution, infusion control, assisting living care, active or passive rehabilitation exercises, etc. Through virtual reality, the communication between medical staff-patient-family-others is further enhanced.

### Prognostic Judgment Based on Big Data

Patients in the intensive care unit are critically ill and change rapidly. Real-time prognosis judgment based on big data has important reference significance for the selection of treatment strategies, especially in places where medical resources are scarce or even lacking, ending ineffective treatment in time is important for saving money. It is of great significance to provide medical resources and treat more patients worthy of treatment. At the same time, it has important reference value for the communication between medical staff and their families.

### Performance Monitoring, Self-diagnosis and Alarm of Medical Equipment

The high utilization rate of instruments and equipment in the intensive care unit plays an important role in maintaining the life of patients. Once failure or performance degradation occurs, the treatment effect will be seriously jeopardized. The management of instruments and equipment based on the Internet of Things technology will help to discover potential hidden dangers in a timely manner, and at the same time, combined with AI, it will give treatment suggestions or reminders, which can prevent major adverse events in time.

### The Role of Intelligent Intensive Care Unit in Clinical Study Activity

The heterogeneity among critically ill patients is high, which greatly hinders the development of clinical research. Based on big data and machine learning, the researchers believe that the ICU clinical information system can improve patient solutions and allocate medical resources, while prompting the entry point of clinical research, and through multidisciplinary cooperation, including clinicians, data engineers, machines learning experts, statisticians, epidemiologists, and other professionals to make the most of ICU data and get clinically meaningful findings [31]. In particular, multi-center research and real-world research in the field of critical care medicine can greatly improve the feasibility and quality of research with the help of intelligent technology.

## Conclusion

The intelligent intensive care unit is a new critical patient management platform gradually developed under the premise of increasing patient demand, technological progress and concept update. The platform integrates the current advanced detection technology, communication technology, analysis technology, and intelligent technology. Based on the results of big data research, systematic, three-dimensional, and comprehensive management and assistance for wards, patients, and staff are carried out, to avoid hidden risks caused by human factors, improve the overall service quality, and make accurate judgments on patients' conditions and prognosis. The future trends of the platform should be more user-friendly. Through the integration of the platform, various personnel will be connected in real time, and ultimately the purpose of improving patient outcomes and optimizing medical resources will be achieved.

## Data Availability

All data generated or analyzed during this study are included in this published article and its supplementary information files.
